# Depression-, Pain-, and Health-Related Quality of Life in Patients with Systemic Lupus Erythematosus

**DOI:** 10.1155/2022/6290736

**Published:** 2022-05-05

**Authors:** Nathalie E. Chalhoub, Michael E. Luggen

**Affiliations:** Division of Rheumatology, Allergy, and Immunology, University of Cincinnati College of Medicine, Cincinnati, Ohio, USA

## Abstract

**Objectives:**

A significant number of patients with systemic lupus erythematosus (SLE) have depression, and many are untreated. We aim to assess the frequency of moderate to severe depression (MSD) in a multiethnic group of SLE patients with different sociodemographic backgrounds, identify modifiable factors associated with depression, and determine the impact of depression, disease activity, damage, cognitive function, and pain severity on health-related quality of life (HRQoL).

**Methods:**

Ninety-nine patients with SLE were evaluated in a cross-sectional study. Sociodemographic data, Beck Depression Inventory (BDI II), SLE disease activity index (SLEDAI-2K), SLICC Damage Index (SLICC-DI), pain severity (10 cm visual analogue scale), cognitive function (Automated Neuropsychologic Assessment Metrics (ANAM)), and the physical (PCS) and mental (MCS) component scores of the Short Form Health Survey (SF-36) were recorded. Bivariate analysis identified potential associations of relevant variables with BDI II and SF-36. Regression analysis determined independent correlates with MSD, PCS, and MCS.

**Results:**

Over 50% of subjects (50.5%) were African-American, 37.1% had a family income of ≤$20,000, and 31.3% had MSD. In the bivariate analysis, family income, SLEDAI-2K, cognitive function, and pain severity were associated with MSD. Using binary logistic regression, SLEDAI-2K and pain severity remained independently correlated with MSD (*p* = 0.004). In the multiple linear regression analysis, pain severity was the only independent correlate of PCS (*p* < 0.0001), while cognitive function and BDI II were the main factors associated with MCS (*p* = 0.020 and *p* < 0.0001, respectively).

**Conclusion:**

Pain severity and disease activity are associated with MSD in our unique population, are potentially modifiable, and deserve further attention in the clinic. Depression and pain significantly affect HRQoL and should be aggressively managed.

## 1. Introduction

Systemic lupus erythematosus (SLE) is a chronic autoimmune disease that affects multiple organ systems. Depression is among the most common neuropsychiatric manifestations of SLE. A recent meta-analysis of 69 studies reported a depression pooled prevalence of 35% [1]. Depression in SLE patients has been shown to adversely affect health-related quality of life (HRQoL) and increase work disability [2–4]. The causes of depression in SLE remain unclear, and various sociodemographic and disease-specific factors have been identified [5, 6]. However, conflicting results have been reported in part because of methodologic issues, differences in metrics, and failure to account for all potentially important covariates [1, 7, 8]. Patients with SLE report higher levels of cognitive difficulties, depression, pain, and fatigue compared with healthy subjects [6, 9, 10]. The precise nature of the relationships among these manifestations is unclear, but depression is undoubtedly playing an important role as both cause and effect. In particular, the interplay of cognitive dysfunction and depression and the influence of pain and other intrinsic disease factors on depression and HRQoL warrants further investigation. Understanding depression and its determinants may provide potential targets for intervention to improve HRQoL and health outcomes in SLE patients.

We have evaluated a multiethnic population with a majority of African American patients and a significant percentage of patients of low socioeconomic status. Only two other studies have evaluated depression in a predominantly African American population that by Heiman et al. who identified depression as a strong correlate of low medication adherence among a population-based cohort of African American individuals with SLE but who did not explore the determinants of depression [11] and that by Falsinnu et al. who evaluated a similar population but whose focus was on pain [12]. The population (Georgians Organized Against Lupus (GOAL)) cohort studied by Heiman et al. is the only one to date with significant numbers of economically disadvantaged subjects (47.4% living below the poverty level) [11].

In this study, we aim to (1) assess the frequency of moderate to severe depression (MSD) in a multiethnic population with different educational and economic backgrounds; (2) identify modifiable factors associated with depression; and (3) determine the impact of depression, disease activity, damage, cognitive function, and pain severity on HRQoL in SLE patients.

## 2. Materials and Methods

Patients with SLE fulfilling the 1997 American College of Rheumatology revised criteria for the classification of SLE [13] were recruited from the Rheumatology Clinic at the University of Cincinnati Medical Center which serves primarily the inner-city poor and from the faculty practices of members of the Division of Rheumatology which receive referrals from community- and university-based primary care physicians as well as subspecialists. The study was approved by the University of Cincinnati Institutional Review Board and commenced in 2011.

After obtaining informed consent, all patients had detailed sociodemographic data collected and were evaluated for depression with the Beck Depression Inventory II (BDI II) [14]. Moderate depression was defined as BDI II ≥ 20 and severe depression as BDI II ≥ 29 [15]. Disease activity was assessed with the SLE Disease Activity Index (SLEDAI-2K) [16] and SLE damage with the SLICC Damage Index (SLICC-DI) [17]. Pain severity was evaluated on a 10 cm visual analogue scale, and cognitive function was determined by the Automated Neuropsychologic Assessment Metrics (ANAM), a symbol-based computerized testing program measuring multiple cognitive domains and previously validated for use in patients with SLE [18–22]. The ANAM4 (Vista LifeSciences, Parker CO) was employed. The total throughput score (TTS) of the ANAM was used as the summary measure of cognitive function, with TTS being the average of the total number of correct responses divided by the time required for those responses for 8 separate cognitive domains. HRQoL was assessed by means of the 36-item Short Form Health Survey (SF-36) [23]. The SF-36 physical component score (PCS) and mental component score (MCS) were recorded on a scale of 0-100 with higher scores indicating better outcomes [23]. Prednisone and immunosuppressive use as well as use of antidepressants and opioids were captured.

### 2.1. Statistical Analysis

Bivariate analyses using the chi-square test and Spearman's correlation were used to identify possible associations of various demographic, disease, and treatment variables with BDI II and SF-36. Binary logistic regression was utilized to determine independent correlates of these variables with MSD (BDI ≥ 20). Candidate independent variables for the logistic regression were chosen on the basis of the bivariate associations (*p* < 0.10). Multiple linear regression was utilized in a forward stepwise fashion to identify independent variables affecting the physical and mental components of SF-36, PCS and MCS. Variables not normally distributed were first transformed by ranks for regression analyses. Analyses were conducted using IBM SPSS Statistics (Version 25).

## 3. Results

In total, 99 patients were evaluated. The sociodemographic data is outlined in Table 1. Mean age was 46.4 (±12.1) years with a female preponderance (93% female vs. 7% male). The study population was comprised of 50.5% African-Americans, 46.5% Caucasians, and 3% either Asians or Hispanic. A total of 43.4% were married, 63.6% had some college education (39.4% with ≥4 years) while 36.4% had not attended college. Family income below or equal to $20,000 per year (approximately the 20^th^ percentile of household income in the US in 2011 when the study commenced and comparable to the US Federal Poverty Level for a family of four at that time) was found in 37.1%. The mean disease duration was 10.6 (±6.8) years. Mean SLEDAI-2K scores were 5.1 (±4.7) indicating mild to moderate disease activity, and mean SLICC-DI scores were 2.4 (±2.1) as shown in Table 2. The mean pain score measured on a 10 cm visual analog scale (VAS) was 3.5 (±2.4) with 44% of patients having moderate to severe pain (≥4.0/10). MSD was identified in 31.3% of patients. PCS mean score was 38.7 (±9.8), and MCS mean score was 43.2 (±11.3). Prednisone was received by 57.1% of whom 12.5% (7.1% of all patients) were on a dose greater than or equal to 20 mg, 68.4% were on hydroxychloroquine, and 41.8% were on other immunosuppressive medications (Table 2). All patients had to complete multiple questionnaires and the ANAM test which can take up to 45 minutes of sustained concentration in a single sitting. This largely excluded patients with severe disease or those who were unstable. In this way, the influence of transient flares or severe organ system dysfunction was mitigated.

A BDI II score with a cutoff of 14 has been used in previous studies to define patients with depression [1, 7]; in our study, using this definition, the prevalence of depression was 53.5% (53/99 patients), and MSD was found in 31.3% of the total population (31/99). A total of 96 patients had their antidepressant use history recorded, of whom 30 had MSD. We noted that 56.7% of MSD patients were on treatment with antidepressants while 43.3% were not.

Chi-square analysis of sociodemographic factors showed the presence of MSD to be significantly associated with family income (*p* = 0.01). There were no apparent associations between MSD and the patients' educational level (*p* = 0.09), ethnicity (*p* = 0.54), marital status (*p* = 0.50), high-dose prednisone (*p* = 0.51), opioid (*p* = 0.20), hydroxychloroquine (*p* = 0.71), or alternate immunosuppression use (*p* = 0.39) (Table 2).

Spearman's correlation identified significant correlations between BDI II score and cognitive function, SLEDAI-2K, and pain severity (Table 3). Using binary logistic regression, SLEDAI-2K scores and pain severity were found to be independent correlates of MSD in patients with SLE. For every increase in the SLEDAI-2K score by 1, the odds ratio increased by 0.2 (OR = 1.2, 95% CI [1.066, 1.407], *p* = 0.004), and every increase in pain severity by 1 cm (on the 10 cm VAS) increased the odds ratio by 0.4 (OR = 1.4, 95% CI [1.129, 1.885], *p* = 0.004). Patients' educational level, family income, and cognitive function were not independently associated with BDI II (*p* values of 0.447, 0.200, and 0.252, respectively).

Patients' HRQoL was assessed by means of the SF-36. Using Spearman's correlation, age, BDI II, and pain severity were found to be significantly correlated with the SF-36 physical component, PCS (*p* = 0.037, *p* < 0.0001, *p* < 0.0001, respectively), while no association was identified between PCS and SLEDAI-2K, SLICC-DI, or cognitive function. MCS was significantly associated with BDI II, SLEDAI-2K, SLICC-DI, cognitive function, and pain severity but not with age (Table 4).

Multiple linear regression analysis with PCS as the dependent variable identified pain severity to be the only variable that is significantly associated with the variance in PCS (*β* = −2.245, 95% CI [-2.991, -1.499], *p* < 0.0001). We noted that 27.6% of the variance in PCS is due to pain severity and that for every increase in the pain level by 1 cm, there is a decrease of 2.245 units on the PCS score. Age and BDI II, even though strongly correlated with PCS on Spearman's correlation, were not identified as independent correlates of PCS using multiple linear regression analysis. The considerable correlation of pain with depression has undoubtedly contributed to mitigating the effect of BDI II on PCS in the multiple linear regression model. Pain severity was a stronger correlate, and after taking it into account in the model, the BDI II score did not contribute much. On the other hand, multiple linear regression using MCS as the dependent variable identified TTS and BDI II to be the main variables associated with MCS. For every increase in BDI II by 1, MCS decreases by 0.657 (*β* = −0.657, CI [-0.801, -0.514], *p* < 0.0001), and for every increase in TTS by 1 (improvement in cognitive function), MCS increases by 0.024 (*β* = 0.024, CI [0.004, 0.044], *p* = 0.020). Depression is a component of MCS and may explain much of its variance. However, when BDI II was removed from the model, TTS was retained (*p* = 0.018) while pain severity and SLEDAI-2K were identified as additional possible independent correlates (*p* = 0.01 and *p* = 0.006, respectively).  Table 5 outlines the variables that were retained by the multiple linear regression model after being applied in a forward stepwise fashion to identify independent variables affecting PCS or MCS.

## 4. Discussion

In this study, we sought to determine the frequency of depression in a cohort of SLE patients of diverse ethnicity and differing socioeconomic status and to identify factors associated with depression with a particular focus on clinical and treatment parameters which were potentially modifiable. We also investigated the impact of depression, disease activity, damage, cognitive function, and pain severity on HRQoL.

In a review and meta-analysis by Moustafa et al., a total of 23,386 patients were studied and had a depression pooled prevalence of 35% for all included studies and of 39.9% for studies that utilized BDI as the depression metric [1]. Zhang et al. in another meta-analysis found a pooled prevalence of depression using BDI (with a cutoff of 14) of 39% also with a 95% confidence interval of 29-49% but with considerable heterogeneity among studies (*I*^2^ = 88.2%) [7]. The prevalence of depression in our patient population using the BDI II with a cutoff of 14 was 53.5%, of whom 58.4% had MSD (31.3% of total population).

Our study prevalence of depression is overall comparable, albeit somewhat higher than that reported previously. This may be related to the rather unique nature of our study population (50.5% African American, 37.1% with annual household income of ≤$20,000) or perhaps the smaller size of our patient population made the frequency estimate less precise. Our reported MSD prevalence (31.3%) is however comparable to a study by Heiman et al. of predominantly African American patients where moderate or severe depressive symptoms were self-reported in 34.6% of African American SLE patients using the 9-item Patient Health Questionnaire [11]. Overall, it is clear that depression affects a significant proportion, if not the majority, of patients with SLE in a multiethnic population.

One of the most important findings in our study was that 43.3% of patients with MSD were not prescribed antidepressants. This would suggest that symptoms of depression in our cohort were either inadequately recognized or else they were felt to be a natural consequence of the disease activity which did not require additional treatment beyond that for the lupus. Similar to our findings, several previous studies have emphasized the lack of recognition and treatment of depressive symptoms in SLE [10, 24, 25]. In two studies, the rate of antidepressant treatment was less than 10% [10, 24]. And, in a third study by Karol et al., only 49% of patients with moderate or severe depressive symptoms were prescribed antidepressants, and many were not receiving optimal dosages [25]. This highlights the need for those caring for patients with lupus to screen for depression, to familiarize themselves with the diagnosis and management of depression in SLE and to consider referral to mental health professionals when depression is severe or unresponsive to standard treatment.

SLE-related pain may have many different causes with approximately 50–90% of patients with SLE reporting pain in different locations, mainly in the musculoskeletal system [26, 27]. A number of studies have suggested that pain is closely associated with depression in SLE [9, 25, 26, 28–31]. Waldheim et al. found that patients with SLE scoring higher degrees of pain were burdened with more fatigue, anxiety, and depression compared to patients with lower levels of pain who did not differ significantly from the general population [26]. And Karol et al. found that pain was an independent correlate of depression (together with only self-reported health status) in a group of 127 clinic patients using a multivariable model [25]. Falasinnu et al. in their predominant African-American population found that patients with depression were more likely to report higher pain intensity in their unadjusted model, but not in the multivariable model using Patient Reported Outcomes Measurement System (PROMIS) adult short forms (SF) to assess depression [12]. The reason for this apparent disagreement is unclear but may be related to the instruments used and the focus on pain as the primary outcome variable.

We similarly identified pain severity as a potentially modifiable correlate of depression in patients with lupus. In our patient population, 88.8% of patients complained of some pain and 44% had moderate to severe pain (≥4.0/10 on VAS). Pain correlated closely with depression in the bivariate analysis and, in multivariable logistic regression, pain was a potent independent correlate of MSD. Pain severity was also correlated with both the PCS and MCS of the SF-36 and, when adjusted for other covariates, remained an independent associated factor of both. Therefore, managing pain more effectively could possibly improve depression. Pain in SLE requires further investigation in order to better understand its multiple dimensions and their interactions. And the treatment of pain *per se* deserves more attention in an optimal management plan for patients with lupus.

Cognitive dysfunction in SLE has been previously investigated in multiple studies with approximately 20-80% of SLE patients experiencing some level of cognitive dysfunction, depending on the definition used. Cognitive function and depression are clearly interrelated. Impaired cognitive function can give rise to depression, and depression is frequently characterized by changes in cognition, including distorted thinking patterns, poor concentration, impaired problem-solving, and reductions in working memory [32]. Both may be the result of other disease related factors such as inflammatory mediators, organs system dysfunction, or medications. Unravelling these interactions may provide important clues to the pathogenesis of both. Few studies have included the assessment of cognitive function in the evaluation of depression [33–36]. The impact of depression on cognitive function was suggested in a multicenter inception cohort of 111 patients with SLE where depressed patients had significantly poorer performance in several cognitive domains of the ANAM test [33]. SLE patients with cognitive dysfunction may also experience higher levels of depression according to Nantes et al. who showed that 60% of cognitively impaired patients had significant levels of depressive symptoms [34]. On the other hand, a study by Calderon et al. described a limited role for depression in cognitive impairment in SLE patients and attributed impairments in sustained attention and spatial working memory to yet-unknown disease-intrinsic factors [35]. And, while recently published longitudinal studies found patients with cognitive dysfunction to exhibit clinically significant depression, the mechanistic link between the two remains not fully elucidated [37, 38]. Our study is one of only a few to attempt to quantitate cognitive function and correlate it with depression while suggesting that other factors such as pain and disease activity could be contributing to the relationship between the two. This definitely warrants further investigation in future longitudinal studies. While we identified a significant correlation between the BDI II scores and TTS scores of ANAM in bivariate analyses, cognitive dysfunction was not found to be an independent correlate of depression in our logistic regression analysis but pain and disease activity were. One explanation for our results could be related to the overlapping attributes and collinearity between pain, depression, and cognitive dysfunction as shown in the study by Lillis et al. [36]. In the latter study, the interplay of pain, depressive symptoms, and cognitive dysfunction in SLE was investigated utilizing mediation modeling [36]. The association between pain and cognitive dysfunction was explained or mediated by sleep disturbance and depression symptoms in a sample of patients with SLE, even after controlling for a number of demographic, medical, and SLE-related treatment and disease covariates [36]. While the lack of correlation between cognitive dysfunction and depression in our study could be related to the association of pain to depression, our analysis revealed TTS to be significantly correlated with MCS of the SF-36 in the bivariate correlation, and in multiple linear regression, TTS and BDI II contributed independently to the model. This result further highlights the strong interplay of cognitive dysfunction and mental health in SLE patients and underscores the need to better understand the interaction between those factors when assessing and managing the larger symptom burden of SLE (depression, pain, cognitive dysfunction, HRQoL outcomes, etc.)

SLE-related disease activity as measured by the SLEDAI-2K was a significant correlate of BDI II scores in this study. In addition, SLEDAI-2K scores were also significantly correlated with MCS and were an independent contributor to MSD in logistic regression. While some authors have determined SLE disease activity as a potential risk factor for the presence and severity of depression in patients with SLE [39–43], others found that SLE disease activity was not associated with depression [5, 10, 44]. Differences in study populations, instruments used, definitions of depression, and study design may account for some of the disparities. However, the longitudinal study by Huang et al. of incident cases of depression is most instructive [5]. They found that while current disease activity as measured by the SLEDAI (SELENA version) was marginally associated with the development of depression, the higher the scores of the SLEDAI, the stronger the correlation that was found [5]. Most studies have reported on patients with minimal to mild activity because only those patients can readily participate. This limits the spectrum of disease activity and may limit the power to detect an association. And, in a recent prospective study of 682 patients, McCormick et al. found that disease activity as measured by the SLAM was significantly and independently associated with depression [45]. There appears to be a significant association between disease activity and depression. For this reason, as well as others, low disease activity or remission should be the ultimate goal.

In our bivariate analysis, prednisone use was not associated with MSD. Previous studies have been inconsistent with respect to the association of depression and steroid use in SLE. Higher-dose prednisone (≥20 mg daily) was identified as an important independent risk factor of depression by Huang et al. [5]. In their univariate analysis, Karol et al. identified moderate/severe depressive symptoms to be associated with prednisone doses of 7.5 mg or more but the association did not reach statistical significance in multivariate analysis [25]. Similar to our findings, Kozora and colleagues found no significant association between prednisone dose and depression [9]. However, we had very few patients receiving higher doses of prednisone (only 7 patients on ≥20 mg/day) which may have limited our ability to detect a difference if one did exist. And, given the cross-sectional nature of this study, it would be difficult to identify such an association if disease activity was closely correlated with depression.

HRQoL is one of the most important outcome measures in assessing patients with lupus. A systematic review of 13 studies including 1279 SLE patients found that SLE patients have lower scores in all SF-36 dimensions [46]. Hence, identifying factors that affect the various components of HRQoL is key in determining modifiable targets that would positively impact SLE patients. In our study, the BDI II score was found to be correlated to both PCS and MCS using bivariate analysis and to MCS in multiple linear regression. The strength of this relationship is very significant and suggests the possibility that MCS could be significantly improved if it were possible to improve depression. This relationship has also been reported by several other investigators [2, 47–50].

Our study has several limitations. The smaller sample size of our cohort precludes definitive exclusion of certain associations between MSD and some of the sociodemographic variables such as educational level and certain clinical variables such as high dose prednisone use, disease duration, and damage index. And we did not compare our results to a cohort of patients with another rheumatologic disease, such as rheumatoid arthritis, which would have allowed us to investigate whether our findings were unique to SLE patients. Finally, ours was a cross-sectional study. The only way to tease apart the complex interrelationships among these variables and to identify true predictors is to perform a rigorous prospective study. However, the main goal of our study was to detect potentially important and modifiable risk factors associated with depression and impaired quality of life in a multiethnic population that deserve further investigation.

In summary, a significant number of patients with SLE have depression, and many are untreated. This clearly underscores the need for heightened awareness, improved diagnosis, and more aggressive treatment of depression in SLE. Our study also found that pain severity and disease activity are associated with the development of moderate to severe depression. These correlates are potentially modifiable and deserve further attention in the clinic. Finally, depression and pain significantly affect HRQoL and must be aggressively managed if the physical and mental health of patients with lupus are to be improved.

## Figures and Tables

**Table 1:** Sociodemographic characteristics of the patients.

Variable	All patients	Moderate-severe depression	No or mild depression	*p* value
Age	46.4 (12.1)	46.4 (12.6)	46.4 (12.0)	0.83
Ethnicity				
Caucasian (%)	46.5	41.9	48.5	
African American (%)	50.5	51.6	50	0.54^∗^
Other (%)	3.0			
Married (%)	43.4	48.4	41.2	0.50
Education (>12 yrs) (%)	63.6	51.6	69.1	0.09
Poverty (%)	37.1	54.8	28.8	0.01

^∗^A *p* value of 0.54 was found when computing the chi square test of MSD vs. non-MSD in Caucasian vs. non-Caucasian patients.

**Table 2:** Clinical characteristics of the patients.

Variable	All patients	Moderate-severe depression^a^	No or mild depression	*p* value
Disease duration (yrs)	10.6 (6.8)	9.6 (7.4)	11.1 (6.5)	0.19
BDI-II^b^	16.7 (11.5)	30.9 (8.3)	10.3 (5.3)	<0.0001
SLEDAI-2K	5.1 (4.7)	7.7 (6.3)	3.9 (3.2)	0.002
SLICC-DI	2.4 (2.1)	2.6 (1.9)	2.3 (2.2)	0.23
Pain severity^c^	3.5 (2.4)	5.0 (2.2)	2.8 (2.2)	<0.001
Prednisone (%)	57.1	54.8	58.2	0.75
≥ 20 mg/day	7.1	9.7	6.0	0.51
Hydroxychloroquine (%)	68.4	71.0	67.2	0.71
Immunosuppressive (%)	41.8	35.5	44.8	0.39
Opioid use (%)	25	33.3	21.2	0.20
Antidepressants use (%)	34.4	56.7	24.2	0.002
SF-36 PCS^d^	38.7 (9.8)	33.7 (7.5)	41.0 (9.9)	<0.001
SF-36 MCS^d^	43.2 (11.3)	32.0 (8.7)	48.3 (8.3)	<0.0001

^a^Moderate depression is defined as BDI II ≥ 20 and severe depression as BDI II ≥ 29. ^b^Beck Depression Inventory II. ^c^Pain severity (on 10 cm visual analogue scale, with 10 being the worst). ^d^PCS is the physical component score, and MCS is the mental component score of the 36-item Short Form Health Survey (SF-36). Scores are measured on a scale of 0-100 with higher scores having better outcomes.

**Table 3:** Spearman correlation of clinical variables relative to BDI II scores.

Variables	Spearman (*ρ*)	*p* value
Age × BDI II	0.047	0.646
TTS∗×BDI II	**-0.289**	**0.004**
SLEDAI − 2K × BDI II	**0.370**	**<0.0001**
SLICC − DI × BDI II	0.126	0.215
Pain severity × BDI II	**0.494**	**<0.0001**
Disease duration × BDI II	-0.137	0.193

^∗^The total throughput score (TTS) of the ANAM test is the summary measure of cognitive function; it is the average of the total number of correct responses divided by the time required for those responses.

**Table 4:** Spearman correlation of clinical variables relative to PCS and MCS.

Variables	Spearman (*ρ*)	*p* value
Age × PCS	**-0.210**	**0.037**
BDI II × PCS	**-0.364**	**<0.0001**
SLEDAI − 2K × PCS	-0.154	0.129
SLICC − DI × PCS	-0.087	0.394
TTS × PCS	0.130	0.203
Pain severity × PCS	**-0.486**	**<0.0001**
Age × MCS	-0.057	0.573
BDI II × MCS	**-0.739**	**<0.0001**
SLEDAI − 2K × MCS	**-0.320**	**0.001**
SLICC − DI × MCS	**-0.241**	**0.016**
TTS × MCS	**0.372**	**<0.0001**
Pain severity × MCS	**-0.365**	**<0.0001**

**Table 5:** Factors associated with the physical and mental component scores, PCS and MCS, based on multiple linear regression.

	Variables	*β* coefficient	Std. error	95% CI	*p* value
PCS	Pain severity	-2.245	0.375	-2.991, -1.499	<0.0001

MCS with BDI II in the model	BDI II	-0.657	0.072	-0.801, -0.514	<0.0001
	Cognitive function	0.024	0.010	0.004, 0.044	0.020

MCS without BDI II in the model	Pain severity	-1.270	0.481	-2.225, -0.315	0.010
	SLEDAI-2K	-0.655	0.231	-1.114, -0.196	0.006
	Cognitive function	0.032	0.013	0.006, 0.059	0.018

## Data Availability

Data is available upon request.
